# Settler Research in Indigenous Country

**DOI:** 10.1177/10778004251390015

**Published:** 2025-11-06

**Authors:** Vicki Chartrand

**Affiliations:** 1 Bishop’s University, Sherbrooke, QC, Canada

**Keywords:** settler research, Indigenous intellectual sovereignty, community-engaged scholarship, collaborative methodology, witnessing, MMIWG2S, relational ethics, decolonial research, land-based knowledge

## Abstract

Settler research in Indigenous country continues to emerge as problematic scholarship, often complicit in an invasive and extractive knowledge production. Drawing on the *Unearthing Justices* project—a collaborative research initiative that shares and showcases Indigenous-led initiatives for the MMIWG2S communities—this article examines the ontological and methodological possibilities for settler research. Foregrounding a practice of witnessing that is relational, embodied, and land-based, the article proposes this approach as a generative intervention into conventional settler research. While acknowledging the inherent tensions and incommensurability of settler presence, this article argues that settler research cannot stand outside colonial systems; instead, it must be reshaped through accountable collaborations that advance Indigenous intellectual sovereignty and community-defined justice. Rather than offer a prescriptive methodology, it invites researchers to stay with the complexities, contradictions, and responsibilities of research in Indigenous country.

*“That which the tree exhales, I inhale. That which I exhale, the tree inhales.”*

Graveline (1998, p. 57)

## Introduction

On Hwy 17B, just outside the city known as Sault Ste. Marie, the words “THIS IS INDIAN LAND” appear in bold white letters on the Garden River train bridge. It was the fall of 1973, a group known as the “original six,” guided by the wisdom of their ancestors, gathered under the cover of night to create an act of (re)presencing (Hopkin, 2023). I remember passing that bridge as a child on family visits, feeling a mix of fear and fascination. Even then, I had already absorbed the national narratives that those words unsettled (Valaskakis, 2005)—narratives that continue to haunt the settler imagination. As it was then, and as it is now: this is Indigenous country.^1^

What does it mean to say this is Indigenous country? Indigenous country is not confined to the borders delineated by settler cartography or the plots of land reserved by the Indian Agent. It is present in every urban, rural, and reserve landscape and in the many spaces and relations that settlers have long ignored through centuries of land theft, resource extraction, and Indigenous erasure. As Native-Land.ca visually and conceptually highlights, the land was originally inhabited very differently around Indigenous lifeworlds, ways of knowing, and the relations between sentient and non-sentient beings. Today, as Mohawk scholar Audra Simpson (2011, pp. 107–108) argues, Indigenous country exists as “spaces of Indigeneity that are framed by settler regimes.” It is situated within a complex web of settler colonial^2^ relations shaped by enduring colonial structures, contested sovereignties, and competing narratives of belonging and presence.

Settler research in Indigenous country is deeply implicated in these contested terrains. It is entangled with the complex spaces, relationships, and tensions that haunt the colonial imaginary. Research has long served as a tool of colonial expansion, used to categorize, control, and dispossess Indigenous peoples and lifeworlds while obscuring settler accountability. It is widely acknowledged that Indigenous peoples in settler states, as elsewhere, are over-studied, their voices are appropriated, altered, or ignored, and that research serves the ends of the researchers, often within colonial projects (Pacific Association of First Nations Women et al., 2005; Quebec Native Women, 2012). The First Nations’ principles of Ownership, Control, Access, and Possession [OCAP] (First Nations Information Governance Centre, 2014) describe settler research as inherently political and contested, one in which Indigenous people are rarely consulted about what information is collected, who gathers it, who maintains it, and who has access to it. Indeed, entire populations have been colonized through the logics and hierarchies of empirical (imperial) and violent knowledge production (Appadurai, 2000; Deloria, 1969). Having encountered critique and claims to legitimacy, the question remains: how can, or should, settler research in Indigenous country take shape?

To attend to this question, I map the trajectory of the *Unearthing Justices* project—a collaborative research initiative developed to create a living digital repository (unearthingjustices.ca) of Indigenous-led initiatives for the missing and murdered Indigenous women, girls, and Two-Spirit (MMIWG2S)^3^ people. *Unearthing Justices* is a three-tiered, community-engaged research project that culminated from long-term and ongoing collaborations that sought to document, share, and showcase the tremendous groundswell and critical mass of community energy in response to the ongoing gendered and racialized violence.^4^

The first stage of the project involved a 17-day, 10,000 km cross-country road trip to Indigenous communities and reserves to discuss and document the stories and insights of local activities and initiatives. Follow-up meetings and discussions took place online, particularly during the COVID-19 pandemic. The second stage consisted of a national media scan of publicly available news and social media sources related to various Indigenous grassroots initiatives addressing MMIWG2S. This scan focused on Indigenous-led, non-governmental, and unfunded efforts, resulting in a resource collection of over 1,000 initiatives, activities, events, and projects across the land. The collection was made publicly available, and community members were invited to add, modify, or remove entries. The third and final stage involved reviewing 118 transcripts and over 100 evidence-gathering testimonies from family members, survivors of violence, experts, and Knowledge Keepers of the National Inquiry into Missing and Murdered Indigenous Women and Girls (2019). Collectively, the Unearthing Justice Project aimed to conceptualize a broad and nuanced understanding of Indigenous grassroots anti-violence organizing and mobilization as part of the larger MMIWG2S movement.

This three-tiered approach provides a multidimensional understanding of MMIWG2S mobilizations, highlighting the complexities and relational dimensions of activities carried out across the land, and tracing and making connections between people and actions (see also Hunt, 2013; Peters, 1998). The cross-country road trip reveals local, intimate community responses to violence; the national media scan exposes the broad scale and diversity of organizing efforts across geographies; and the testimonies from the National Inquiry situate this work within lived experiences of state-based harm and systemic violence and racism. As Carol Martin (NIMMIWG, 2018, pp. 186–196) testifies, Indigenous women are bombarded by laws, policies, and procedures that work against them at every level of their lives, where often the only recourse is refusal.

These layers of documentation—local, national, and testimonial—sought to do more than catalog initiatives; it was a method to paint a picture of a movement and a living landscape of refusal, care, and self-determination. Attending to this mobilization as a settler researcher, however, is not a neutral act. It demands an attention to the responsibilities, limitations, and inheritances embedded in any attempt to trace, represent, or give shape to Indigenous-led movements. In response to these settler tensions, the project was grounded in a methodology shaped by invitation, witnessing, and ongoing collaborations. Overall, the project emerged through a relational commitment and accountability that sought to advance Indigenous intellectual sovereignty. I remain aware of the pressing questions of legitimacy that shape settler research in Indigenous country (see also Gombay & Palomino-Schalscha, 2018). Rather than justify, legitimate, or otherwise resolve these tensions, this article remains within their incommensurability.

## Settler Research

Critiques of settler knowledge production have challenged and shifted possibilities for research. Said (1978/1993) demonstrates how research serves as an imperial mechanism that silences, erases, appropriates, and rewrites cultural, historical, and political worldviews and representations. Haraway (1988) defines these erasures as the “God trick,” where research claims the ability to see everywhere while originating from nowhere. As a scientific project, research is a ritual of storytelling that constructs the “gaze of the text/author as universal and research as un-situated, neutral, ahistorical, acultural, and an unquestionable production” (Duran & Duran, 1995; Gordon, 2008; Wahab, 2005, p. 30). In its production, however, research shapes and limits what counts, what can and cannot be said and done, and by whom, while erasing place, belonging, and identity in the process. Cultural and Indigenous scholars have pulled back this veil to expose research’s racialized and geopolitical incursions while offering different possibilities for rethinking the relations of research.

A critical engagement with modern knowledge production reveals the subtle yet pervasive ways in which colonial structures and logics continue to shape research. For example, Audra Simpson (2014), a Mohawk scholar, explores how settler colonialism structures knowledge production to exclude Indigenous political thought, sovereignty, and refusal from academic discourse. Peter Cole (2004), of St’át’imc and Celtic heritage, critiques institutional ethical research practices, noting a frequent disconnect between ethical rhetoric and actual practices that prioritize bureaucratic compliance and processes over thoughtful and respectful engagement with communities. Gina Starblanket (2019), of Cree and Saulteaux descent, underscores how the concept of community in research can homogenize Indigenous peoples into narrow frameworks of identity and culture. Even research centered on colonialism in Indigenous lives can perpetuate colonial relations and restrict Indigenous freedom from it (Alfred & Corntassel, 2005). These works show that research is not a neutral or benevolent practice, but how it continues to shape knowledge through colonial structures and relations.

Even when research seeks to be “critical” or “emancipatory,” it can inadvertently reproduce research hierarchies through subtle cultural dominance (Kincheloe et al., 2008), humanitarian justifications (Césaire, 1955), cognitive imperialism (Walker, 2003), and white savior tropes (Batacharya & Wong, 2018). Cabot (2016) argues that the representational practices of ethnography aimed at “empowering” marginalized voices continue to utilize tragic tropes that reinforce passivity and dependence. Similarly, de Leeuw and Hunt (2018) of Tłaliłila’ogwa contend that reflexivity in research can inadvertently reinforce white centrality rather than disrupt it. These scholars demonstrate that even when research takes on a liberatory stance, colonial tropes and relations persist within the methods, reaffirming entrenched relations of whiteness, paternalism, and imperialism.

Given these embedded colonial structures, rethinking research in Indigenous country necessitates an ontological shift—one that shifts away from detached, universalizing methods toward relational, land-based and accountable research. Indigenous approaches to research emphasize writing from “very specific somewheres” (Coburn et al., 2013, p. 335). Unlike the “God trick,” these “somewheres” view knowledge and its creation as situated, relational, and embedded in a set of responsibilities to all creation (Aveling, 2013; Kovach, 2021; Tuhwai Smith, 2021). Lauren Tynan (2020), a Trawlwulwuy woman from Tebrakunna country, observes that knowledge is not produced by those conducting research; rather, it is shared with us by country through all its earthly and ancestral relations. As they walk through the suburbs of Dharawal/Yuin, the author notices the sky erupting with white sulfur-crested cockatoos and hears the call of “the-sis” as they fly overhead. Tynan now understands thesis or “the-sis” as a relational insight shared by country, which emerges through observing natural and ancestral relations in their everyday world. This insight positions knowledge not as a product of research but as a gift shared through reciprocal engagement with land and kin. Country is not merely the backdrop for inquiry or something on which research occurs, but is a place of knowing and learning—an active, living entity of constellations of relationships that shapes and informs possibilities for learning, understanding, and knowing.

The term “kinship” is compelling for reframing research as part of a consortium of accountable relations rather than as a discrete, individualized process. Kim TallBear (2014), a citizen of the Sisseton-Wahpeton Oyate, extends this relational kinship framework to research itself, contending that methodologies should not reinforce settler categories of identity but should instead be structured around kinship—understood not merely as biological descent or rigid categories commonly found in scholarly classification, but as an active set of responsibilities to land, ancestors, and community. Chris Andersen (2009), Métis, refers to this idea as density—the complex range of Indigenous experiences, knowledges, and relationships, including their collective, epistemological, and cultural dimensions. “Density” signifies the depth and layered nature of Indigenous existence, which is often flattened or simplified in dominant representations. In this view, identity is not solely tied to individual or community characteristics but is rooted in family, collectivities, ancestry, land, and cultural connections, among other things. This density of kinship encompasses the connections of accountability among diverse entities, including research that forms only a small part of a broader relational field. TallBear and Andersen caution that research should not reanimate Indigeneity in ways that reinforce settler narratives and institutions while erasing actual Indigenous intelligence, kinship, and sovereignty. Kinship is instructive for accountable research, grounded in mutually reinforcing responsibilities and right relations in research and knowledge production.

Within Indigenous frameworks, a research approach acknowledges multiple material worlds are constituted by human and non-human entanglements, which perform and shape reality in diverse ways orbiting seasons and places (Deloria, 1973). Shifting research to land-based, relational, and ephemeral qualities entails uncertainty, complexity, and staying; aspects that conventional scholarly methods often fail to capture or conduct. As Wendy Rose (1994), of Hopi and Miwok descent, shows, knowledge production is a delicate mapping of the barely visible affinities and connections, all while navigating a fine line between engaging and appropriating experiences. Like a river, the process unfolds through the relational forces that shape it; structured yet seldom predictable, meandering through unexpected insights and connections. It navigates blind spots and uncertainties, reflecting on the ever-present tensions, conflicts, and complicities of settler research in Indigenous country.

## War Pony

The seeds of the Unearthing Justice Project first germinated when I met Gladys Radek in 2009 while I was working at a women’s shelter in Quesnel, British Columbia—a predominantly white settler town boundaried by reserves and near the Highway of Tears (Hwy 16), where over 75 Indigenous women have been disappeared or murdered (Sterritt, 2023). Gladys is Gitxsan Wet’suwet’en, a family member and grassroots activist. At that time, she was on one of her seven cross-country walks supporting the families of the missing and murdered. Through these walks, Gladys and other volunteer walkers traverse thousands of kilometers, encountering countless individuals and family members along their travels. As they move from town to town, they carry the names and stories of those they meet, raising awareness and connecting families across the land. I crossed paths with Gladys again a year later at a conference in northern British Columbia in 2010, where she was the keynote speaker. Two years later, in 2012, I saw her once more in Ottawa at a Take Back the Night march on Parliament Hill, where I learned that she was organizing a fifth walk across Canada. This walk would begin in Sydney, Nova Scotia, and end in Prince Rupert, British Columbia, covering roughly 7,500 km over 100 days. By this third encounter, I was invited into this life’s work with Country; an invitation that shaped our work and drew us into relation.

The months leading up to the national walk involved many volunteers and were intensely focused on planning and organizing, including securing funding, coordinating logistics, engaging the public, and mobilizing support. This involved fundraising efforts, hosting events, recruiting volunteers, strategic meetings with parliamentarians and dignitaries, detailed planning and mapping of the walk, media outreach, volunteer coordination, and logistical tasks such as acquiring supplies and maintaining vehicles. From the outset, it became clear that these walks were far more than simple awareness-raising efforts. The behind-the-scenes work revealed the profound labor, commitment, strategy, and care that sustained a national movement. As much as it was about “raising awareness,” the work built ongoing connections, movement, growth, and mobilizations.

Being invited into this work was not simply a matter of participation. It was an entry into a relational field shaped by supportive work and commitments. An invitation from community is not a conventional research approach that generates a problem or question and steps in to find answers or solutions. Instead of starting with research, it arises within a relational field of connections, responsibilities, and a shared ethos. In this context, relationality is not merely a concept but a lived practice, one that is learned and developed through reciprocity, obligation, shared experiences, co-existence, cooperation, and social memory (Moreton-Robinson, 2000, p. 16). Through relationality, the intimate details and understanding of the work’s density, and your relationship to it, begin to surface. Invitations into community are neither permanent nor open—they are contingent, fragile, and shaped by where and how you belong, as well as the roles and responsibilities that accompany being present in spaces that are not inherently your own. An invitation draws us into spaces and relations, establishing ethical and affective connections that shape our lives and work.

These connections are most visibly expressed and embodied through War Pony—the vehicle that accompanies walkers on their journey. Adorned with images of missing and murdered Indigenous women, girls, and Two-Spirit people, gifted by their families, War Pony is more than just a vehicle; it serves as a national Indigenous icon to keep the memories and spirits of the missing and murdered alive, connecting families and sparking conversations across the country (Morritt-Jacobs, 2019). The idea for War Pony was inspired by Gladys and Bernie Williams Poitras, Golden Spruce Woman of the Haida Gwaii Nation, who co-organized the Walk4Justice movement—a series of cross-country walks aimed at raising awareness and justice for MMIWG2S families. Over the years, War Pony, in its various forms, has traveled thousands of kilometers, including a significant journey from northern British Columbia to Gatineau, Quebec, for the closing ceremony of the National Inquiry into MMIWG. More than just a vehicle, War Pony is a mobile archive, a site of memory, and a living testament to lives stolen by colonial violence. Covered in photographs as it journeys across the land, it transforms public spaces into sites of remembrance, bringing families together and compelling settlers to confront the tensions between the “true, north, strong and free” (Mawani, 2007) and the endemic violence against the life-givers of this land.

War Pony signifies a relational presence in the places it travels through. On our road trip, upon arrival in Indigenous communities or reserves, many residents would approach War Pony to look at the images posted on the car—some of whom they knew and many others who also had someone in their lives who had been disappeared or murdered. In contrast to the distant and voyeuristic gaze War Pony would receive in settler communities, Indigenous communities routinely supported the walkers, donating food and gas, providing information, offering places to stay, and sharing their stories of loved ones. War Pony and the walkers embody witnessing as method—not just recounting stories of loss, but carrying them forward in ways that create visibility, connect families, and re-presence the memories and spirits of the many who have been stolen from their families and communities. The cross-country walks map stories across the land, transforming routes into pathways of remembering and honoring. The emphasis on movement—walking as a form of action, grieving, and visibility—parallels how settler research in Indigenous country cannot be static but fluid, present, and connected.

It was only through the unfolding connections, an invitation to contribute, and a commitment to the work that made the *Unearthing Justices* possible, giving shape to something that might otherwise have remained unseen. The work began not with the research, but with the density of the labor, tracing it across the land through the intimate knowledge of one national walk within an otherwise sea of activities. In this sense, research was taking shape through relationships, responsibilities, and experiences of the movement itself. When shaped by invitation, research witnesses and engages with the full weight of the work—not as an abstract problem to solve but as a living, entangled process of responsibility, care, and action. A research approach engaged in invitational relations makes visible the intricate layers of labor in sustaining movements like these. Through accountable relationality, research does not merely document but participates in the ongoing, contingent, and affective work of justice, making the unseen dimensions of mobilization and resistance more deeply felt and understood. By engaging in these intimate spaces, research does not pretend to be objective, but learns to be a part of something.

## Constellations and Campfires

The idea for the *Unearthing Justices* project was born from these early and long-term collaborations to create a living repository of the collective movement and density of work. The following year, Gladys and I set out on a cross-country road trip, retracing the path of her many walks of connecting with the many families and communities entrenched or touched by the murders and disappearances. Rather than focusing on the pain or suffering, this project sought to document the stories, struggles, and actions grounded in their day-to-day needs and strategies for loved ones and for each other. By documenting the initiatives of family and community members, the project sought to capture how these activities inform an inclusive and grounded approach to justice and provide a broader understanding of their experiences and contributions. The stories gathered on this journey were not just testimonies of loss, but blueprints for action, revealing the strategies and wisdom of those engaged in long-term justice work.

Research by settlers in Indigenous country is fraught with challenges of representations and epistemic assumptions over what is seen, valued, and legitimized. Outside research typically has goals and objectives external to the community—namely, the accumulation of knowledge for advancement (Blaney, 2003). As Seneca scholar Mishuana Goeman (2013, pp. 19–20) notes, traditional research methods reflect colonialism’s “violent spatial legacy” of “imperial imaginations,” which erase and negate traditional practices, stories, and connections to the land. The author advocates for a (re)mapping approach to reconfigure, re-articulate, and represent Indigenous experiences and understandings of land and territory in textual, virtual, and lived contexts (see also Goeman, 2008; Hunt & Stevenson, 2016; Iralu, 2021; Llanes-Ortiz et al., 2019; Martineau & Ritskes, 2014). Remapping and reconfiguring research to capture a vital yet overlooked essence of knowledge and understanding necessitates an intimate connection with the land and people.

As part of an intimate awareness, the project aimed to collect the stories and intelligence of communities—not only to document but shaping the very nature of the project. Driving War Pony, we visited reserves, suburbs, and urban centers, connecting with friends, families, and community members on the lands known as Manitoulin Island (Ontario), Thunder Bay (Ontario), Winnipeg (Manitoba), Regina (Saskatchewan), Kelowna (British Columbia), Vancouver (British Columbia), Quesnel (British Columbia), Edmonton (Alberta), and Saskatoon (Saskatchewan). We documented our travels through hundreds of pictures, capturing the mosaic of landscapes and people we encountered. While field notes were sparse, the visual documentation helped map the terrain, and the textual documentation that did occur was in the form of journaling rather than “field” experiences (Sanjek, 2019). This mode of documenting our travels reflects a different, embodied relationship to knowledge gathering and production through place-based thought (Watts, 2013), connecting stories to the land. At night, we camped out by trees and waterways, campgrounds, and, at times, parking lots. This road trip was by far the most intense and magical. Guided by relationships and moments rather than a fixed methodology, the project responded to what emerged, shaped not just by what was gathered, but by *how* it was gathered. Every kilometer, town, and day brought a different energy and awareness. The stretches of highway, long silences, countless conversations, constellations, campfires, and being present in spaces of grief, struggle, resistance, and care is a responsive research engagement.

The land occupies an ontological framework for knowing, shaping, experiencing, and relating to the world and each other (Coulthard, 2010, p. 79). The landscapes are filled with stories and spirits carried through land-based activities and commemorations, some of which we visited on our travels. These commemorations are not passive acts of remembrance but a presencing—a returned and enduring presence complicated by its relationality to settler colonialism (Martineau & Ritskes, 2014). From the loss of lives, child apprehensions and disappearances, assaults and abuses, removal of life-sustaining supports and resources, and displacements from vital connections, the magnitude of loss, grief, hardship, disconnections, and struggle present are, as Toni Morrison (1988) might say, unspeakable. If colonization remains the condition that makes violence possible, the activities of the families and communities are a counter-colonial recourse of visibility and colonial dismantling. This was expressed through stories of community care and coming together through initiatives like search toolkits, guides for working with the police and media, drone searches, dragging rivers, offering accommodation or paying for a hotel room, building banners and signs, providing gas and supplies, offering food, and fundraising, among many more. These activities highlight the collective intergenerational nature of this work, spanning over 50 years of advocacy. They structure the significance of land-based knowledge in research and re-create the foundations of knowing through a physical presencing (Johnson & Larsen, 2013, p. 10) whereby knowledge and relationships are organized, lived, and shared through the land.

The locations of these land-based activities and commemorations are important for their associated purposes, particularly in areas where loved ones were last seen, places they often frequented, sites of ceremonies, parks, public spaces, and areas with high visibility. Given the incredible feats achieved to bury the violence against MMIWG2S people, land-based presencing is central to justice. These grassroots activities include community patrols and watch groups, search toolkits and apps, anti-violence workshops, healing circles, safe rides, and memorials and walks to raise awareness, among many others. Indigenous families and communities have long been involved in actively addressing the systemic intersections of gendered and racialized violence. Their work has been critical in advancing what communities need to advance justice, including accountability, advocacy, caring, celebrating, ceremony, compassion, educating, growing, healing, helping, honoring, mourning, nurturing, protecting, remembering, safety, shelter, sharing, and supporting. The same rhythms of movement that shape the daily actions of advocacy, care, and remembrance also shape the possibilities for justice as a live and continuous practice.

Tracing these activities on our road trip and in our conversations, immersed the research into the physical and temporal rhythms of people, land, and movement. Unlike traditional research, which often relies on observation and analysis from a relatively fixed positionality, the journey gently and forcefully pulls you to look at things in ways you would not have thought or considered otherwise. In other words, remapping research methods through movement produces possibilities for thinking about the activities and relations carried out in specific places and making previously unseen connections between them. Research is inseparable from ourselves and the collectives and entities we engage in, growing and changing through our encounters and activities—both known and yet to be known.

## Tobacco and Stone

On our trip, we brought gifts of tobacco and stones. As it was explained to me, the tobacco, or aseema, among the Anishinaabe, is a cultural symbol used to seek guidance, share information, and express gratitude. As Gladys explained, the tobacco was not a gift for the recipient, but for the creator to will the words and truth to come forward. The stones, in contrast, were drawn from my own life, as I had collected and carried them to many places over the years. In this gift economy, the essence of the gift creates a set of relationships (Kimmerer, 2013, p. 28). Offering tobacco and choosing a stone before each discussion signaled a relational entry point that established conditions and possibilities for dialogue. On one hand, this exchange gestures toward a point of connection and the sharing of ideas (Chilisa, 2012: 222). On the other hand, it reflects the tensions that arise when settler research enters unfamiliar spaces and knowledge systems.

Witnessing in research involves being present in the density of our own and others’ lives, appreciating the weight of what has been shared, and then moving differently as a result. Deloria & Wildcat (2001, p. 12) note that we are intimately connected, related, and dependent in our density—the complex personhoods, layered identities, and political landscapes that shape how we come into relation. Witnessing requires “presence, repetition, and responsibility . . . that we show up, over and over again, in ways that affirm and sustain life” (Simpson, 2017, p. 151). The *Unearthing Justices* project is a method of concrete, embodied, and relational practice of presence, accountability, and responding. It is traveling, listening, holding space, absorbing silences, driving War Pony, sharing food, choosing a stone, and showing up again the next day. It is how research moves—through roads, relations, and repetition, not through detached methodologies.

Throughout the project, witnessing reinforced the density of complex personhood (Gordon, 2008)—the strength, the softness, the strategic refusals, and the fragility that shape people’s lives and work. The conversations revealed how Indigenous activities and struggles that are systematically unwitnessed within settler states are characteristically taking place from their homes, at the kitchen table, outside of institutional structures. More than a place of conversation, the kitchen table is a site where memory, story, grief, laughter, and strategy are shared (Palacios, 2016). Gaertner (2015) describes the kitchen table as more than a research prop but as a dynamic site of debate, dialogue, and political unpacking. The kitchen table is a signifier for the critical and strategic dialogue that emerges from these intimate and personal spaces and the pivotal conversations that provide intelligibility for action.

Shawn Wilson (2020), a member of the Opaskwayak Cree Nation, highlights how these dialogues are best understood through storying—a method that connects and makes visible the obscured and abstracted aspects of life. Wilson describes knowledge as a living entity within a web of relationships, including those with ancestors, the land, and future generations. Unlike the linear arguments and discrete categories of traditional research, storying weaves together different strands of experience, insight, and context, making it possible to see how seemingly unrelated aspects of life or the ephemeral are interwoven.

Although many of these shared stories took place at the kitchen table, others occurred at a picnic table, beside a fire or a memorial, or in the backyard, basement, or at a restaurant. The physical location shaped the stories and silences. At Crab Park, a waterfront memorial site, conversations were deeply reflective and focused on the marches and remembrance. Outside, times spent and lost with loved ones would surface, followed by silences when the wind would touch our faces. Indoor restaurants, conversations focused almost exclusively on advocacy and action: logistics, funding and policy action. These spaces reveal that no single setting is a neutral “field site,” instead, shaping how each infinite story unfolds. The kitchen table is not an abstract backdrop upon which research occurs, but a methodological centerpiece for thought and analysis—a co-constituted and epistemic space that shapes the conversations, all depending on who is at the table, how they arrived, and whether they are prepared to stay.

Throughout the conversations, we discussed the various approaches and experiences of the work, the impacts it has had on them, their families, and communities, as well as the challenges they encountered. Many reflected on the shared experiences of loss, pain, and displacement that have shaped their lives and collective memory. Although contrived, the interview performance also gave legitimacy to the project and some weight to the conversations. Many family members worked hard to make their voices visible, public, and heard, and the interview helped validate and amplify this invisible work. The interview performance is both a barrier and a bridge. It carried the weight and performance of being recorded, but also being witnessed, giving visibility to stories often ignored.

As stories, interviews are not passive inlets of information but actively shared knowledge. The idea of the interview, however, with consent forms, video recording equipment, and a research presence, imposes relations and forces awkward or suspicious conversations. Throughout the project, that layered tension, simultaneously invitational and complicated, was ever-present and continually mediated. During our travels, it became clear that the exchanges and conversations were only possible due to Gladys’s presence and long-term, ongoing relationships (Episkenew, 2009; Weber-Pillwax, 2001, p. 170). The interviews took place through a brokered trust that had been built through years of advocacy, walks, and many shared stories. Throughout the conversations, Gladys would, at times, relate how we had met and the purpose of the project or share her own experiences in relation to the stories told. Most often, she sat silently and listened. It also became clear that brokered relations are as important as the stories being shared (Aikau et al., 2015).

The *Unearthing Justices* project is not a retelling or interpretation of data, but a situating of these stories in the wider currents of collective experience and memory of strategy and struggle. It is a living archive shaped by the people and movement. As argued by Martineau and Riske (2014, p. iii), research can be a way to “reclaim the literal, discursive and imaginative terrain of decolonial struggle” and “trace evolving articulations of Indigenous resurgence that break the vow of silence and invisibility.” Settler research in Indigenous country involves knowing what research can offer, what it cannot, where to step up, and where to step out. It calls for”

diverse work that seeks to better cope with our self-evidently more-than-human, more-than-textual, multisensual worlds . . . offering an escape from the established academic habit of striving to uncover meanings and values that apparently await our discovery, interpretation, judgement and ultimate representation. (Vannini, 2015, p. 318)

Ongoing vigilance in relation to the fragility of your participation is necessary.

## Unearthing Justices

Settler research in Indigenous country is, invariably, a colonial encounter. It is already imprinted by the histories that shape these interactions before you even arrive. Setting out on this project, although I could not destabilize these colonial tensions, I could situate myself within the context of my life and work—a soon-to-be mother, a researcher, an invited accomplice, and with access to the resources of an academic institution designed for extracting, evaluating, organizing, disseminating, and archiving knowledge. Where I could not step outside the structures that shape research, we can repurpose their instruments to access, redirect, and redistribute them in support of documenting, sharing, and amplifying.

Just as settler research and its institutions have levied a violent mode of knowledge production against Indigenous communities, research can also be used to shift some of these trajectories. The *Unearthing Justices* project was established to redirect knowledge production concerning MMIWG2S people away from a state-centric focus on inaction and absence toward grassroots presence. Although government accountability is important, this orientation overshadowed the significant mass of Indigenous grassroots work and organization. Unlike state-driven and media narratives that document MMIWG2S movement as “raising awareness” activities, the realities of the work are best understood through Indigenous grassroots efforts, those who are at the heart of the struggle having spent decades mobilizing, walking, remembering, honoring, supporting, advocating, and resisting the continued erasure of MMIWG2S lives (Million, 2013). The *Unearthing Justices* project aims to digitally collate, share, and showcase how communities are central to dismantling violence in largely unnoticed ways.

Community-led initiatives show how Indigenous grassroots responses create protective and supportive networks in the face of systemic failure. For example, Safe Passage in Portage LaPrairie offers women and girls traveling on the isolated road a safe place to stay. Neechi Rides provides free and safe transportation for Indigenous people in and around Winnipeg. Sister Watch in Vancouver brings visibility and support to urban areas through community wrap-around services. Thunderbird Operation mobilizes online crowdsourcing to gather and circulate information about missing persons. Community patrols and watch groups span the nation, building relational safety through presence, support, and a sense of accountability that is not based on enforcement, but on kinship. These efforts are not auxiliary to justice—they are justice, enacted through care and the refusal to wait for state intervention.

From on-the-ground community efforts to digital and literary activism, Indigenous grassroots responses to the MMIWG2S violence are not just calls for justice; they are practices and possibilities for justice in themselves, within a local and relational context (see also Craft, 2013; Monture-Angus, 1999). This work is critical in advancing what communities need in the moment and the context in which it is needed. The daily actions of advocacy, care, and remembrance highlight that justice is a lived, active, and continuous practice. Rather than locate justice in some abstract institution or legal application, *Unearthing Justices* decenters the state and explores justice in its diverse locations, people, relations, and applications. Justice here takes on relational, generative, and purposeful qualities that shape and are shaped by one’s belonging, resources at hand, sheer will, and love. Such a conception of justice is operationalized on what materially and currently exists in communities, particularly given the long-term and ongoing state and public absence and neglect in relation to the murders and disappearances.

This project seeks more creative and collaborative understandings of and approaches to justice, exploring possibilities for connecting people, sharing models, and identifying areas where resources are most needed. Locating this work as justice is a reframing of the activities in a way that centers the so-called margins as exceptional spaces and brings attention and focus to the communities. Running with the current of the families and communities, the project insists that the struggle for justice must remain visible, carried forward in ways that are community-driven and accessible. Documenting this presence through a live repository, the project aims to counteract state-focused resourcing, fleeting reports, and archival erasures. Dian Million (2013, p. 54) describes this as a “felt archive,” offering “a narrative that appeals as a history that can be felt as well as intellectualized” and honoring the complexity of lived experience under colonial conditions. The living repository is a collection of individual activities and experiences, but is located within a larger collective context. By creating a public repository that communities can engage with, the project also seeks to break down some silos and that the knowledge shared returns to those who offered it.

As this project shows, settler research has access to mainstream resources and has something to offer in the exchange of knowledge, information, insights, time, and energy, whether that is amplifying voices, mobilizing knowledge, finding information, providing research materials and insights, public education, funding, acknowledging, or listening—all active parts in the method of witnessing. Academics, government, and the public have much to learn from the families and communities central to organizing and mobilizing around the MMIWG2S people. By drawing attention to grassroots solutions, *Unearthing Justices* seeks to amplify the work and visibility of MMIWG2S families and communities, reduce some of the barriers between them, promote networks of resources and support, and bring the focus and resources to the communities where they belong.

## Conclusion

Settler research in Indigenous country is not a typified kind of scholarship. It is an undertaking that grows you in unexpected ways—the kind of growth that not only challenges the legitimacy of your involvement but also your place as an inhabitant in a shared world shaped by deeply ignored relations. As a settler researcher, I know my awareness and perception of the life worlds at play in Indigenous country are limited. This project was not to produce knowledge about Indigenous lives, but to honor, share, and showcase what is already there.

This article invites a reorientation of settler research, not through certainty or solution, but through a method of witnessing that is accountable, relational, and sustained. Rather than seeking to resolve the inherent tensions of settler research, this project holds space for its incommensurability, while repurposing its articulation through an awareness of the politics of presence and witnessing the density of people’s lives. This article does not offer a methodological prescription for settler research (see Ahenakew, 2016), but rather a rethinking of the relations of research with a deep regard for the lives and lifeworlds involved. A relational accountability to research decenters the researcher, prioritizes commitment, shares resources, and advances Indigenous intellectual sovereignty and self-determination.

I get it—and I get it wrong—all the time. Through this work, I have learned that a collectivity of people’s lives, including my own, is carried in the decisions we make and the ideas and activities we project into this world through our research and beyond. We cannot afford to get it wrong, but we do. By documenting and tracing MMIWG2S and the community-led efforts to remember them, the project traces the story of a movement and, like the bold letters on the Garden River train bridge, its presence.

## Notes

1. The term “Indigenous country” is adapted from the concept “Indian country”—a term that originated in U.S. federal law (18 U.S.C. § 1151), but its use has been adopted and discussed by various Indigenous scholars, activists, and communities to reflect their own interpretations, understandings, and critiques (Deer & Kronk Warner, 2019).

2. When I say colonial, it does not suggest that it is not a stable category, one of a passive or unchanging nature, but pluralistic, with multiple experiences, and actively negotiated within real and perceived, visible and invisible arrangements (Mignolo, 2007).

3. Two-Spirit is a shorthand acronym for gender and sexually diverse people who identify outside gender binaries and heteronormative sexuality. This shorthand is not intended to erase the significance of these fluid and complex expressions (Lezard et al., 2021).

4. The blanket terms “community” and “Indigenous grassroots” can be vague and homogenizing, potentially suggesting an overall coherence, harmony, or even utopia (e.g., see Anderson, 2016; Rose, 1992). Although they may essentialize, these terms serve as useful placeholders for identifying collective actions and organizing.

## References

[bibr1-10778004251390015] Ahenakew C. (2016). Grafting Indigenous ways of knowing onto non-Indigenous ways of being: The (underestimated) challenges of a decolonial imagination. International Review of Qualitative Research, 9(3), 323–340. 10.1525/irqr.2016.9.3.323

[bibr2-10778004251390015] Aikau H. K. Arvin M. Goeman M. Morgensen S. (2015). Indigenous feminisms roundtable. Frontiers: A Journal of Women Studies, 36(3), 84–106. 10.5250/fronjwomestud.36.3.0084

[bibr3-10778004251390015] Alfred T. Corntassel J. (2005). Being Indigenous: Resurgences against contemporary colonialism. Government and Opposition, 40(4), 597–614. 10.1111/j.1477-7053.2005.00166.x

[bibr4-10778004251390015] Andersen C. (2009). Critical Indigenous studies: From difference to density. Cultural Studies Review, 15(2), 80–100. 10.5130/csr.v15i2.2039

[bibr5-10778004251390015] Anderson K. (2016). A recognition of being: Reconstructing Native womanhood. Canadian Scholars’ Press.

[bibr6-10778004251390015] Appadurai A. (2000). Grassroots globalization and the research imagination. Public Culture, 12(1), 1–19. 10.1215/08992363-12-1-1

[bibr7-10778004251390015] Aveling N. (2013). “Don’t talk about what you don’t know”: On (not) conducting research with/in Indigenous contexts. Critical Studies in Education, 54(2), 203–214. 10.1080/17508487.2012.724021

[bibr8-10778004251390015] Batacharya S. Wong Y.-L. R. (2018). Sharing breath: Embodied learning and decolonization. Athabasca University Press. 10.15215/aupress/9781771991919.01

[bibr9-10778004251390015] Blaney F. (2003). Aboriginal women’s action network. In Anderson K. Lawrence B. (Eds.), Strong women stories: Native vision and community survival (pp. 156–163). Sumach Press.

[bibr10-10778004251390015] Cabot H. (2016). “Contagious” solidarity: Reconfiguring care and citizenship in Greece’s social clinics. Social Anthropology, 24(2), 152–166. 10.1111/1469-8676.12297

[bibr11-10778004251390015] Césaire A. (1955). Discourse on colonialism ( Pinkham J. , Trans.; Brydon D. , Ed.). Routledge. 10.4324/9781003101406

[bibr12-10778004251390015] Chilisa B. (2012). Indigenous research methodologies. Sage.

[bibr13-10778004251390015] Coburn E. Moreton-Robinson A. Sefa Dei G. Stewart-Harawira M. (2013). Unspeakable things: Indigenous research and social science. Socio, 2(2), 331–348. 10.4000/socio.524

[bibr14-10778004251390015] Cole P. (2004). Trick(ster) s of Aboriginal research: Or how to use ethical review strategies to perpetuate cultural genocide. Native Studies Review, 15(2), 7–30. https://publications.usask.ca/nativestudiesreview/issues/Vol15No2.html

[bibr15-10778004251390015] Coulthard G. (2010). Place against empire: Understanding Indigenous anticolonialism. Affinities: A Journal of Radical Theory, Culture, and Action, 4(2), 79–83. https://ojs.library.queensu.ca/index.php/affinities/article/view/6141

[bibr16-10778004251390015] Craft A. (2013). Breathing life into the Stone Fort Treaty: An Anishnabe understanding of Treaty One. UBC Press.

[bibr17-10778004251390015] de Leeuw S. Hunt S . (2018). Unsettling decolonizing geographies. Geography Compass, 12(7), Article e12376. 10.1111/gec3.12376

[bibr18-10778004251390015] Deer S. Kronk Warner E. A. (2019). Raping Indian country. Columbia Journal of Gender and Law, 38(1), 31–95.

[bibr19-10778004251390015] Deloria V. Jr. (1969). Custer died for your sins: An Indian manifesto. Macmillan.

[bibr20-10778004251390015] Deloria V. Jr. (1973). God is red: A Native view of religion. Putnam.

[bibr21-10778004251390015] Deloria V. Jr. Wildcat D. R. (2001). Power and place: Indian education in America. Fulcrum Publishing.

[bibr22-10778004251390015] Duran E. Duran B. (1995). Native American postcolonial psychology. State University of New York Press.

[bibr23-10778004251390015] Episkenew J. (2009). Taking back our spirits: Indigenous literature, public policy, and healing. University of Manitoba Press.

[bibr24-10778004251390015] First Nations Information Governance Centre. (2014). Ownership, Control, Access and Possession (OCAP™): The path to First Nations information governance. https://fnigc.ca/ocap-training/

[bibr25-10778004251390015] Gaertner D. (2015). Indigenous in cyberspace: CyberPowWow, God’s Lake Narrows, and the contours of online Indigenous territory. American Indian Culture and Research Journal, 39(4), 55–78. 10.17953/aicrj.39.4.gaertner

[bibr26-10778004251390015] Goeman M. (2008). From place to territories and back again: Centering storied land in the discussion of Indigenous nation-building. International Journal of Critical Indigenous Studies, 1(1), 34. 10.5204/ijcis.v1i1.20

[bibr27-10778004251390015] Goeman M. (2013). Mark my words: Native women mapping our nations (1st ed.). University of Minnesota Press. 10.5749/minnesota/9780816677900.001.0001

[bibr28-10778004251390015] Gombay N. Palomino-Schalscha M. (2018). Indigenous places and colonial spaces: The politics of intertwined relations. Routledge.

[bibr29-10778004251390015] Gordon A. F. (2008). Ghostly matters: Haunting and the sociological imagination. University of Minnesota Press.

[bibr30-10778004251390015] Graveline F. J. (1998). Circle works: Transforming Eurocentric consciousness. Fernwood Publishing.

[bibr31-10778004251390015] Haraway D. (1988). Situated knowledges: The science question in feminism and the privilege of partial perspective. Feminist Studies, 14(3), 575–599. 10.2307/3178066

[bibr32-10778004251390015] Hopkin J. (2023, June 22). “This is Indian Land”: Train bridge commemorated in Garden River. Sootoday.com. https://www.sootoday.com/local-news/this-is-indian-land-train-bridge-commemorated-in-garden-river-7179242

[bibr33-10778004251390015] Hunt D. Stevenson S. A. (2016). Decolonizing geographies of power: Indigenous digital counter-mapping practices on Turtle Island. Settler Colonial Studies, 7(3), 372–392. 10.1080/2201473X.2016.1186311

[bibr34-10778004251390015] Hunt J. (2013). Engaging with Indigenous Australia—Exploring the conditions for effective relationships with Aboriginal and Torres Strait Islander communities. Australian Institute of Health and Welfare, Closing the Gap Clearinghouse. https://openresearch-repository.anu.edu.au/server/api/core/bitstreams/71ad05dd-1b1a-4764-b468-8ba25a77589d/content

[bibr35-10778004251390015] Iralu E. (2021). Putting Indian Country on the map: Indigenous practices of spatial justice. Antipode, 53(5), 1485–1502. 10.1111/anti.12734

[bibr36-10778004251390015] Johnson J. T. Larsen S. C. (2013). A deeper sense of place: Stories and journeys of Indigenous-academic collaboration (1st ed.). Oregon State University Press.

[bibr37-10778004251390015] Kimmerer R. W. (2013). Braiding sweetgrass: Indigenous wisdom, scientific knowledge and the teachings of plants. Milkweed Editions.

[bibr38-10778004251390015] Kincheloe J. L. Steinberg S. R. Denzin N. K. Smith L. T. Lincoln Y. S. (2008). Indigenous knowledges in education: Complexities, dangers, and profound benefits. In Denzin N. K. Lincoln Y. S. Smith L. T. (Eds.), Handbook of critical and Indigenous methodologies (pp. 135–156). Sage. 10.4135/9781483385686.n7

[bibr39-10778004251390015] Kovach M. (2021). Indigenous methodologies: Characteristics, conversations, and contexts (2nd ed.). University of Toronto Press.

[bibr40-10778004251390015] Lezard P. Prefontaine Z. Cederwall D.-M. Sparrow C. Maracle S. Beck A. McLeod A. (2021). MMIWG2SLGBTQQIA National Action Plan: Final report (2SLGBTQQIA Sub-Working Group). Province of Manitoba. https://www.gov.mb.ca/inr/mmiwg/pubs/2slgbtqqia-report-final.pdf

[bibr41-10778004251390015] Llanes-Ortiz G. Castillo R. A. H. Noble B. Hutchings S. (2019). Maya knowledges, intercultural dialogues, and being a Chan Laak’ in the Yucatán Peninsula. In Noble B. Castillo R. A. H. Hutchings S. (Eds.), Transcontinental dialogues (pp. 166–190). University of Arizona Press.

[bibr42-10778004251390015] Martineau J. Ritskes E. (2014). Fugitive Indigeneity: Reclaiming the terrain of decolonial struggle through Indigenous art. Decolonization: Indigeneity, Education & Society, 3(1), I–XII.

[bibr43-10778004251390015] Mawani R. (2007). Legalities of nature: Law, empire, and wilderness landscapes in Canada. Social Identities, 13(6), 715–734. 10.1080/13504630701696351

[bibr44-10778004251390015] Mignolo W. D. (2007). Delinking: The rhetoric of modernity, the logic of coloniality and the grammar of de-coloniality. Cultural Studies, 21(2–3), 449–514.

[bibr45-10778004251390015] Million D. (2013). Therapeutic nations: Healing in an age of Indigenous human rights (1st ed.). University of Arizona Press.

[bibr46-10778004251390015] Monture-Angus P. (1999). Journeying forward: Dreaming First Nations’ independence. Fernwood Publishing.

[bibr47-10778004251390015] Moreton-Robinson A. (2000). Talkin’ up to the white woman: Indigenous women and feminism. University of Queensland Press.

[bibr48-10778004251390015] Morrison T. (1988, October 7). Unspeakable things unspoken: The Afro-American presence in American literature [Lecture transcript]. The Tanner Lectures on Human Values. https://quod.lib.umich.edu/m/mqrarchive/act2080.0028.001/19:2?g=mqrg;page=root;rgn=full+text;size=100;view=text;xc=1;q1=toni+morrison

[bibr49-10778004251390015] Morritt-Jacobs C. (2019, June 4). The journey of Gladys Radek and her fight for human rights. APTN News. https://www.aptnnews.ca/national-news/the-journey-of-gladys-radek-and-her-fight-for-human-rights/

[bibr50-10778004251390015] National Inquiry into Missing and Murdered Indigenous Women and Girls [NIMMIWG]. (2018) The National Inquiry’s consolidated literature review of reports relating to violence against Indigenous women, girls, and 2SLGBTQQIA people. Government of Canada. https://www.mmiwg-ffada.ca/publication/consolidated-literature-review-violence-against-women-and-girls/

[bibr51-10778004251390015] National Inquiry into Missing and Murdered Indigenous Women and Girls. (2019). Reclaiming power and place: Final report of the national inquiry into missing and murdered indigenous women and girls. https://www.mmiwg-ffada.ca/final-report/

[bibr52-10778004251390015] Pacific Association of First Nations Women, Ending Violence Association of BC, & BC Women’s Hospital & Health. (2005). Researched to death: B.C. Aboriginal women and violence. Pacific Association of First Nations Women. https://canadacommons.ca/artifacts/1214561/researched-to-death/1767658/

[bibr53-10778004251390015] Palacios L. (2016). Challenging convictions: Indigenous and Black race-radical feminists theorizing the carceral state and abolitionist praxis in the United States and Canada. Meridians, 15(1), 137–165.

[bibr54-10778004251390015] Peters E. J. (1998). Subversive spaces: First Nations women and the city. Environment and Planning D: Society and Space, 16(6), 665–685.

[bibr55-10778004251390015] Quebec Native Women. (2012). Guidelines for research with Aboriginal women. Quebec Native Women. https://www.faq-qnw.org/wp-content/uploads/2016/11/QNW-2012-Guidelines_for_Research.pdf

[bibr56-10778004251390015] Rose W. (1992). The great pretenders: Further reflections on white shamanism. In Jaimes M. A. (Ed.), The state of Native America: Genocide, colonization, and resistance (pp. 403–423). South End Press.

[bibr57-10778004251390015] Rose W. (1994). The politics of resistance: American Indian fiction, feminism, and literary nationalism. American Indian Quarterly, 19(1), 45–55. https://www.jstor.org/stable/1185652

[bibr58-10778004251390015] Said E. (1993). Culture and imperialism. Vintage Books. (Original work published 1978).

[bibr59-10778004251390015] Sanjek R. (2019). Ethnography in today’s world: Color, class, and culture. University of Pennsylvania Press.

[bibr60-10778004251390015] Simpson A. (2011). Settlement’s secret. Cultural Anthropology, 26(2), 205–217. 10.1111/j.1548-1360.2011.01095.x

[bibr61-10778004251390015] Simpson L. B. (2014). Dancing on our turtle’s back: Stories of Nishnaabeg re-creation, resurgence, and a new emergence. Arbeiter Ring Publishing.

[bibr62-10778004251390015] Simpson L. B. (2017). As we have always done: Indigenous freedom through radical resistance. University of Minnesota Press. 10.5749/j.ctt1pwt77c

[bibr63-10778004251390015] Starblanket G. (2019). The numbered treaties and the politics of incoherency. Canadian Journal of Political Science/Revue canadienne de science politique, 52(3), 443–459.

[bibr64-10778004251390015] Sterritt A. (2023). Unbroken: My fight for survival, hope, and justice for Indigenous women and girls. Greystone Books Ltd.

[bibr65-10778004251390015] TallBear K. (2014). Standing with and speaking as faith: A feminist-Indigenous approach to inquiry. Journal of Research Practice, 10(2), 1–7.

[bibr66-10778004251390015] Tuhwai Smith L. T . (2021). Decolonizing methodologies: Research and Indigenous peoples (3rd ed.). Bloomsbury Publishing Plc. 10.5040/9781350225282

[bibr67-10778004251390015] Tynan L. (2020). Thesis as kin: Living relationality with research. AlterNative: An International Journal of Indigenous Peoples, 16(3), 163–170.

[bibr68-10778004251390015] Valaskakis G. G. (2005). Indian Country: Essays on contemporary Native culture. Wilfrid Laurier University Press.

[bibr69-10778004251390015] Vannini P. (2015). Non-representational ethnography: New ways of animating lifeworlds. Cultural Geographies, 22(2), 317–327. 10.1177/1474474014555657

[bibr70-10778004251390015] Wahab A. (2005). Consuming narratives: Questioning authority and the politics of representation in social science research. Counterpoints, 252, 29–51.

[bibr71-10778004251390015] Walker P. (2003). Colonising research: Academia’s structural violence towards Indigenous peoples. Social Alternatives, 22(3), 37–40.

[bibr72-10778004251390015] Watts V. (2013). Indigenous place-thought and agency amongst humans and non-humans (First Woman and Sky Woman go on a European world tour). Decolonization: Indigeneity, Education & Society, 2(1), 20–34.

[bibr73-10778004251390015] Weber-Pillwax C. (2001). Coming to an understanding: A panel presentation—What is Indigenous research? Canadian Journal of Native Education, 25(2), 166–174.

[bibr74-10778004251390015] Wilson S. (2020). Research is ceremony: Indigenous research methods. Fernwood Publishing.

